# In Vivo Measurements of T2 Relaxation Time of Mouse Lungs during Inspiration and Expiration

**DOI:** 10.1371/journal.pone.0166879

**Published:** 2016-12-09

**Authors:** Lars E. Olsson, Paul D. Hockings

**Affiliations:** 1 Department of Medical Radiation Physics, Translational Medicine, Lund University, Malmö, Sweden; 2 Antaros Medical, BioVenture Hub, Mölndal, Sweden; 3 Medtech West, Chalmers University of Technology, Gothenburg, Sweden; Karl-Franzens-Universitat Graz, AUSTRIA

## Abstract

**Purpose:**

The interest in measurements of magnetic resonance imaging relaxation times, T1, T2, T2*, with intention to characterize healthy and diseased lungs has increased recently. Animal studies play an important role in this context providing models for understanding and linking the measured relaxation time changes to the underlying physiology or disease. The aim of this work was to study how the measured transversal relaxation time (T2) in healthy lungs is affected by normal respiration in mouse.

**Method:**

T2 of lung was measured in anaesthetized freely breathing mice. Image acquisition was performed on a 4.7 T, Bruker BioSpec with a multi spin-echo sequence (Car-Purcell-Meiboom-Gill) in both end-expiration and end-inspiration. The echo trains consisted of ten echoes of inter echo time 3.5 ms or 4.0 ms. The proton density, T2 and noise floor were fitted to the measured signals of the lung parenchyma with a Levenberg-Marquardt least-squares three-parameter fit.

**Results:**

T2 in the lungs was longer (p<0.01) at end-expiration (9.7±0.7 ms) than at end-inspiration (9.0±0.8 ms) measured with inter-echo time 3.5 ms. The corresponding relative proton density (lung/muscle tissue) was higher (p<0.001) during end-expiration, (0.61±0.06) than during end-inspiration (0.48±0.05). The ratio of relative proton density at end-inspiration to that at end-expiration was 0.78±0.09. Similar results were found for inter-echo time 4.0 ms and there was no significant difference between the T2 values or proton densities acquired with different interecho times. The T2 value increased linearly (p< 0.001) with proton density.

**Conclusion:**

The measured T2 in-vivo is affected by diffusion across internal magnetic susceptibility gradients. In the lungs these gradients are modulated by respiration, as verified by calculations. In conclusion the measured T2 was found to be dependent on the size of the alveoli.

## Introduction

Relaxation times T1 and T2/T2* may reflect structural changes of lung tissue caused by physiology, respiration, lung injury or clinical pulmonary disease [1,2,3]. A few examples for T1: there is a small difference between the T1 measured at end-expiration and end-inspiration [4]. T1 of the lung depends on both sex and age [5], and it was recently found that T1 relaxation time was shortened in lung for COPD patients [6].

The relaxation time T2* in lungs has been proven to be sensitive to the structure and physiology of the lungs. It has been shown that in-vivo T2* of the lungs is related to the alveolar size in mouse [7], state of inflation in humans [8] and emphysema in rats [9]. Additionally, T2* is a potential biomarker of smoke and emphysema in humans [10]. It is worth noting that the effect of the state of inflation on T2* is larger than the effect on T1 and also theoretically underpinned [11].

Compared to T1 and T2* less attention has been directed towards T2 relaxation time measurements of the lungs in recent years. However, there is an extensive literature from the 1990’s with particular emphasis on ex vivo studies in isolated lungs [12]. In animal models, changes in T2 relaxation time have been a parameter of interest for radiation induced lung damage [13] and pulmonary edema [14,15]. Alveolar size has been shown to influence the measured T2 of lung ex vivo [16], and other ex vivo experiments have shown that T2 is dependent on lung inflation [1]. Ganesan et al. [17] studied the effect of the inflation pressure on T2 using Hahn echoes in excised rat lungs and showed that T2 was longer in degassed lung than in inflated lung. Similar results were reported using the CPMG technique (Carr-Purcell-Meiboom-Gill) [18]. However, the effect was rather small and no difference was found for intermediate inflation pressures ex vivo [17,19]. It was concluded that water diffusion across the internal magnetic field gradients, which are modulated by the respiration, was responsible for the T2 change [20].

Recently, interest in T2 measurements of the lung has been rekindled. T2 values of the lungs were used to differentiate between various regional patterns of interstitial lung disease [21] and a Hahn prepared spin-echo T2 weighed sequence has been used to assess the alveolar size in human lungs in-vivo [22]. However, T2 measurements of animals have only been performed in vitro on excised organs, which hardly describe the environment in a living and freely breathing animal. In-vivo animal studies play an important role in this context providing models for understanding and linking the measured relaxation time changes to the underlying physiology or disease. At present little is known about how the normal respiration modulates changes in the measured T2 in living animals.

The aim of this study was to measure in vivo T2 relaxation time in mouse at end-inspiration and end-expiration during normal breathing at 4.7 T.

## Materials and Methods

Air and tissue have different susceptibility and the alveolar structure in the lung creates microscopic magnetic field gradients in the tissue. The diffusion of spins through these internal gradients affects the magnetization and as a result the measured T2 relaxation time decreases and be expressed as [23]:

$$\frac{1}{T2}=\frac{1}{{T2}_{int}}+\frac{1}{{T2}_{diff}}$$ (1)

T2_int_ is the intrinsic T2 without diffusion effects and T2_diff_ is the effect of diffusion.

The expected effect of the internal magnetic field gradients on the measured T2 with a Carr-Purcell-Meiboom-Gill (CPMG) sequence can be estimated from [23]:

$$\frac{1}{T2}=\frac{1}{{T2}_{int}}+\frac{\gamma^{2}G^{2}{TE}^{2}D}{12}$$ (2)

where G is the magnetic field gradient experienced by the diffusing protons, with the diffusion coefficient D during the interecho time TE. Note that G includes both the imaging gradients and the susceptibility gradients of the alveolar tissue interfaces. The latter gradient will be modulated by the respiratory process [20].

### Animals

The study was approved by the Local Ethical Animal Review Board (Gothenburg University, 2012/185). Ten male CB57BL/6 (9–12 weeks old; weight 18–22 g) were anaesthetized with isoflurane (~1–2%) inhalation in a 1:1 air oxygen mix. The animals were freely breathing during image acquisition. The animals were terminated immediately after the completion of the experiment by sodium pentobarbital and spine dislocation.

### MR imaging

The experiments were performed on a 4.7 T MR scanner (BioSpec 47/40, Bruker, Ettlingen, Germany) with a BGA12 gradient insert (max strength = 400 mT/m, Ø = 120 mm) and a manually tuned linear polarized transmit/receive coil (Ø = 35 mm).

The mice were anaesthetized and positioned on a dedicated animal bed equipped with a fluid based heating system driven by a thermostat-controlled pump to maintain body temperature (Haake DC 10, Thermo Electron Corporation, Philadelphia. PA, USA). Respiration was tracked by a small pressure sensitive pad on the abdomen connected to a computer controlled monitoring system (Model 1025, SA Instruments Inc, Stony Brook, NY, USA).

The imaging session started with a set of localizers, to ensure that the lungs of the mouse were approximately in the center of the magnet and the coil. Coronal, sagittal and transaxial spin-echo images of the lungs were acquired to outline the basic anatomy of the thorax and serve as localizers for the following imaging.

All mice were scanned with a 2D multi spin echo sequence (CPMG) with inter echo time 3.5 ms and 10 echoes for the T2 measurement. The repetition time was 1000–1200 ms depending on breathing rate, FOV was 30x30 mm^2^, matrix 96x96, bandwidth 100 kHz, and 6 averages were acquired. With the help of the localizer images, a coronal slice of 1.5 mm thickness was located centrally in the lungs, with both lung lobes depicted clearly. Two sets of images were acquired, one at end-inspiration and the other at end-expiration. All details of the sequence are given in Table 1.

**Table 1:** MR imaging protocol. A 2D multi spin-echo sequence for T2 measurement. The repetition time is somewhat dependent on the respiratory rate.

Parameter	Value	Parameter	Value
Sequence	2D MSE	Repetition time, TR	~1000 ms
Field of view	30x30 mm2	Echo time, TE	3.5 (or 4.0)
Matrix	96x96	No of echoes	10
Orientation	coronal	Bandwidth, BW	101 kHz
Slice thickness, SL	1.5 mm	Acquisition time, TA	15 min
No of slices	1	No of excitations, NEX	6

For five of the mice the T2 measurement was repeated using an inter echo time of 4.0 ms keeping all other settings identical. These measurements were also carried out at end-inspiration and end-expiration.

### Evaluation

The image calculations and post processing of the images was performed with in-house software (IDL, Research Systems Inc, Boulder, CO, USA). T2-maps and relative proton density (PD) maps were calculated on a pixel-by-pixel basis. The parameters PD, T2 and noise floor (NF) were fitted to the signals from the multi spin echo images according to Eq (3), with a Levenberg-Marquardt least-squares three-parameter fit:

$$S=PD\;e^{-\frac{TE}{T2}}+NF$$ (3)

On the T2-maps of the thorax the lungs were segmented out with a seeding algorithm. All pixels with T2 values between 1 and 15 ms were assigned to lung tissue. The chosen T2 interval excluded major vessels and other tissue in the lung area from the ROI. A second ROI was manually drawn in muscle tissue. The PD values were read from the corresponding PD map with the coordinates taken from the T2 segmentation. The PD for the lung was normalized to the PD of muscle in each animal.

If not stated otherwise, reported values are given as mean values plus/minus one standard deviation, and the statistical analysis was performed with a paired two-tail student’s t-test.

## Results

**Fig 1:**
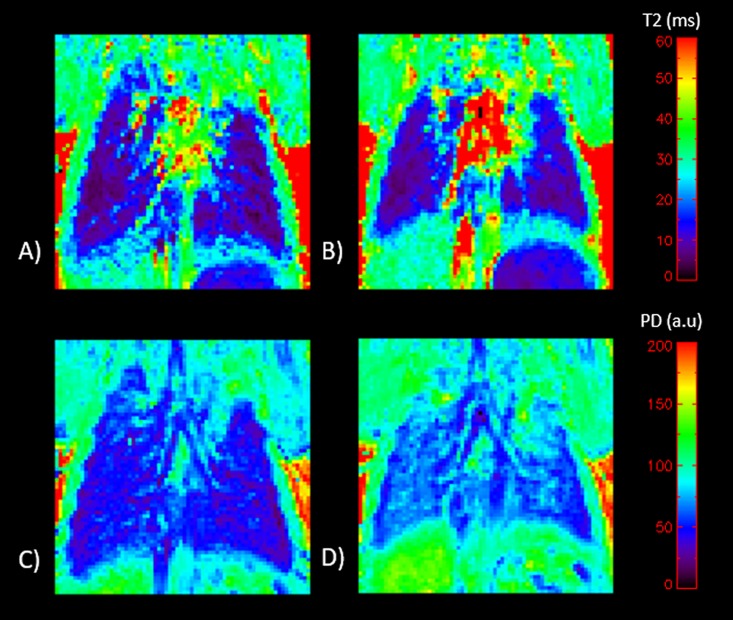
Examples of calculated T2-maps for A) end-inspiration, and B) end-expiration. The T2 values (ms) are indicated on right hand color bar. The corresponding relative proton density maps C) end-inspiration, and D) end-expiration. Relative PD values (PDlung/PDmuscle)*100) are indicated on the right hand color bar. All images are from the same mouse.

One animal was excluded due to a technical problem with the triggering device. For the remaining nine mice it was possible to obtain images of the lungs during both end-inspiration and end-expiration from which T2-maps could be calculated. The T2 map and the corresponding PD-map of the lung obtained during end-inspiration are noticeably larger than the corresponding images obtained during end-expiration of the same mouse. The small change in measured T2 is not perceptible directly from the image, but the change in relative proton density is clearly depicted (Fig 1).

**Fig 2:**
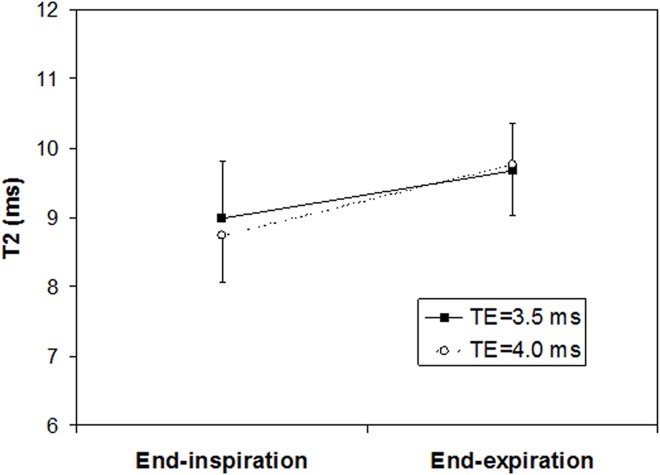
T2 measured during end-inspiration and end-expiration. The difference is significant for both inter echo times (p<0.01). The difference between T2 obtained at different echo times is larger at end-inspiration than end-expiration (NS).

The measured T2 in the lungs was longer at end-expiration than at end-inspiration. Using an inter echo time of 3.5 ms the T2 was 9.7±0.7 ms and 9.0±0.8 ms (p<0.01), for end-expiration and end-inspiration, respectively. With an inter echo time of 4.0 ms the T2 was 9.8±0.7 ms and 8.7±0.7 ms (p<0.01), for end-expiration and end-inspiration, respectively (Fig 2). There was no significant difference between the measured T2 values acquired with different inter echo time. The ratio between T2 measured at end-expiration and end-inspiration was higher for inter echo time 4.0 ms (1.12±0.04) than 3.5 ms (1.08±0.07), but the difference was not significant.

**Fig 3:**
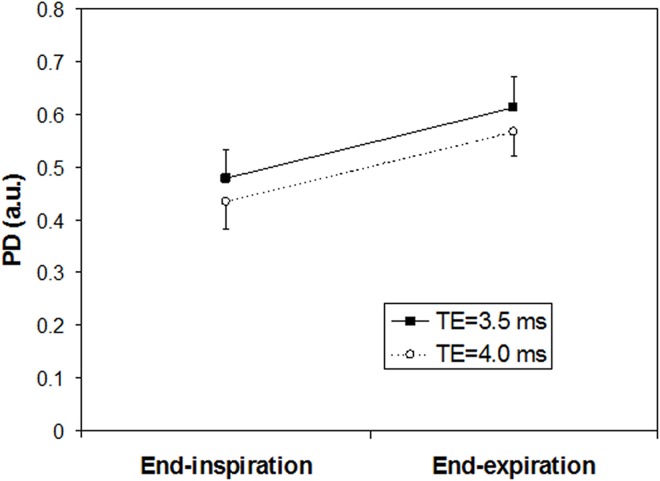
Relative proton densities (from the T2 measurement). The PD is higher at end-expiration than end-inspiration. The difference is significant for both inter echo times (p<0.01).

**Fig 4:**
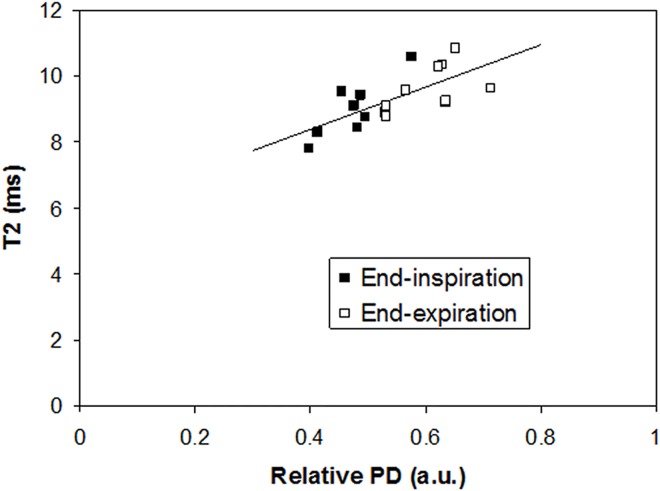
T2 as a function of the relative proton density. T2 increases with increasing proton density (p<0.001, Pearson correlation) r = 0.7.

The relative proton density (lung/muscle tissue) was higher during end-expiration than during end-inspiration. With an inter echo time of 3.5 ms the PD was 0.61±0.06 and 0.48±0.05 (p<0.001), for end-expiration and end-inspiration, respectively. The ratio between PD at end-inspiration and end-expiration was 0.78±0.09. The PD results for inter echo time 4.0 were 0.57±0.05 and 0.43±0.05 (p<0.001), for end-expiration and end-inspiration, respectively. The PD ratio between end-inspiration and end-expiration was 0.76±0.05. The PD seems higher for the shorter inter echo time but the difference is not significant (Fig 3). The measured T2 value increased with increasing PD (p<0.001, Pearson correlation) with r = 0.7 (Fig 4).

For reference the T2 was calculated in muscle tissue. At inter echo time 3.5 ms the T2 was 31.2±2.6 and 31.7±1.5 ms, for end-inspiration and end-expiration, respectively. For inter echo time 4.0 ms the T2 was 31.1±1.3 and 32.3±1.5 ms, for end-inspiration and end-expiration, respectively. No differences were significant.

## Discussion

In this study T2 relaxation time was measured in mouse lungs. To the best of our knowledge T2 measurements for different levels of respiration have not been reported in vivo for mouse lungs previously. We found a small but significant difference between the T2 relaxation time obtained from measured data in end-inspiration and end-expiration. In the mouse lungs the measured T2 was longer at end-expiration than end-inspiration. The animals were not mechanically ventilated and the change between end-inspiration and end-expiration represents the normal respiratory cycle. The effect on T2 was significantly smaller than the effect previously found on T2* [7].

Changes in T2 for different states of inflation have been noted previously for isolated rat lungs, where the T2 increased by almost 100% going from collapsed to degassed lungs. However, the differences in T2 at intermediate inflation pressures were non-significant [17]. Similar results were obtained by other researchers [1,18,24]. The linear relationship between proton density and the measured T2 in this study supports the argument that the changes obtained are dominated by a change in alveolar size. However, we cannot completely exclude that alveolar recruitment could have a minor role [25], even if this process is not studied in detail for small rodents. Theoretically, increasing inter-echo times should increase the sensitivity to alveolar size [23]. This theoretical relationship was recently examined in detail and confirmed experimentally in an ex-vivo model [26]. In this paper we report a non-significant increase in the difference in T2 between end-inspiration and end-expiration with a longer inter-echo time.

We assume that the effect of lung inflation on T2 can be attributed to water diffusion across the internal magnetic field gradients during the inter-echo time [20,23]. An increase in alveoli size during inspiration will induce an increase in the internal susceptibility gradients. The internal gradients can be calculated from Eq 2 if the intrinsic T2 is known. Since it is difficult to measure intrinsic T2 of lung in vivo without the effect of diffusion through field gradients, its value was derived from literature data. Ex vivo degassed lungs are lung tissue without the susceptibility gradients induced by air-tissue interfaces. T2 for degassed rat lungs measured at 2.1 T is 21.8 ms [27]. Tissue T2 generally increases slightly at higher field strengths. Assuming that the magnetic field dependence of degassed lung T2 is similar to that of muscle and not different for rat and mouse [28,29], then the intrinsic T2 is 25 ms for mouse lung at 4.7T. Other input data are echo-time 3.5 ms and the diffusion coefficient D = 1.26 10^−8^ m^2^/s representing the apparent diffusion coefficient in highly vascularized tissue [30]. From Eq 2, the internal gradient was 0.26 T/m for end-expiration and the gradient is increased by 0.016 T/m due to inspiration. Similar large magnetic field gradients of 0.075 T/m were recently found in human lung (approximate alveolar size 200 μm) at 1.5 T [22]. The larger gradients reported here are expected due to the higher magnetic field of 4.7 T. However, every model is a simplification, and the choice of diffusion constant and estimation of intrinsic T2 have large impact on the result. We conclude that the estimated gradients are high, but are consistent with other recent reports.

The ratio between PD at end-inspiration and end-expiration was 0.76–0.78. From the literature we have an end-expiration volume of 0.5 ml and tidal volume of 0.15 ml for mouse [31]. These figures result in an expected PD ratio between end-inspiration and end-expiration of 0.77, in close agreement with our experimental findings.

From the relative proton density (lung/muscle) obtained in this study the approximate alveoli volume fraction can be estimated. The measured proton densities for end-expiration and end-inspiration (0.61 and 0.48) correspond to a volume fraction of approximately 0.39 and 0.52. Volume fraction has been used in empirical models describing the transverse relaxation for heterogeneous media as systems composed of randomly distributed stationary magnetic spheres surrounded by proton spins undergoing unrestricted, isotropic diffusion motion [32]. In this model the 1/T2_diff_ is linearly proportional to air volume fraction of the lung parenchyma. In this study there was a 30% increase of volume fraction from end-expiration to end-inspiration. As a result, one would expect a decrease of T2 by 1.5 ms. This is more than the measured result of 0.7 ms, but in the correct order of magnitude. Additionally, although the major change during inspiration is in alveolar enlargement, there is also a possibility that some degree of alveolar recruitment is involved.

There are several other factors that may influence the measured T2 values of the lungs: molecular oxygen concentration in the alveoli, dissolved molecular oxygen concentration in blood, the oxy/deoxyhemoglobin levels in pulmonary venous and arterial blood, and oxygen concentration within tissue. However, these factors will only be of importance if they fluctuate with the respiratory cycle. Oxygen is exchanged between the alveolar air and the blood through diffusion, which occurs in response to concentration gradients. Under normal breathing conditions these gradients are more or less in steady state. In a study of freely breathing lambs a variation of 2% was found in the oxygen arterial pressure over the respiratory cycle [33]. This variation is a too small to change either the dissolved molecular oxygen concentration or the oxy/deoxyhemoglobin ratio in the pulmonary venous blood significantly enough to affect the T2 [34,35]. A similarly small change in molecular oxygen concentration can be expected in both the alveoli and pulmonary parenchyma during the respiratory cycle, which also is a too small to be of significance for T2 [36,37]. Using the same logic, the molecular oxygen concentration of the pulmonary arterial blood will not be dependent on the respiratory cycle.

Altogether, it can be concluded that the major cause for the change in T2 found in this study can be explained and attributed to the effect of water diffusion across the internal susceptibility gradients modulated by respiration. In conclusion, T2 was found to be dependent on the state of inflation, and thereby the size of the alveoli. To our knowledge, this is the first time the phenomenon is observed in vivo and for the normal breathing cycle. An imaging biomarker for alveolar remodeling could be used to follow progression or potentially regression of emphysema and COPD. This will be the subject for future studies.
